# LinguaPix database: A megastudy of picture-naming norms

**DOI:** 10.3758/s13428-021-01651-0

**Published:** 2021-08-10

**Authors:** Agnieszka Ewa Krautz, Emmanuel Keuleers

**Affiliations:** 1 Department of English Linguistics, University of Mannheim, Schloss EW 274, 68161 Mannheim, Germany; 2 Department of Cognitive Science and Artificial Intelligence, Tilburg University, Warandelaan 2, 5037 AB Tilburg, the Netherlands

**Keywords:** Picture-naming norms, Picture database, Speech onset times (SOT), Familiarity, Visual complexity, Valence, Arousal

## Abstract

The major aim of the present megastudy of picture-naming norms was to address the shortcomings of the available picture data sets used in psychological and linguistic research by creating a new database of normed colour images that researchers from around the world can rely upon in their investigations. In order to do this, we employed a new form of normative study, namely a megastudy, whereby 1620 colour photographs of items spanning across 42 semantic categories were named and rated by a group of German speakers. This was done to establish the following linguistic norms: speech onset times (SOT), name agreement, accuracy, familiarity, visual complexity, valence, and arousal. The data, including over 64,000 audio files, were used to create the LinguaPix database of pictures, audio recordings, and linguistic norms, which to our knowledge, is the largest available research tool of its kind (http://linguapix.uni-mannheim.de). In this paper, we present the tool and the analysis of the major variables.

## Introduction

Pictures are very often utilised as stimuli in psychological and linguistic research. They are used in a wide variety of experimental tasks, such as picture naming, translation, and the visual world paradigm. In a picture-naming paradigm, for instance, participants are asked to name, as quickly and accurately as possible, pictures that are shown in succession on a computer screen, while their reaction times and error rates are recorded. This type of task appears to be very simple, yet it is very useful, as pictures are believed to activate underlying semantic information (Altarriba & Basnight-Brown, 2009). In other words, the picture-naming task allows for drawing conclusions about the way in which semantic information is processed and represented in memory. Given that ‘semantic memory is one of our most defining human traits, encompassing all the declarative knowledge we acquire about the world’, and it is the basis for almost all human activity (Binder & Desai, 2011, p. 1), the importance of pictures as experimental stimuli in psycholinguistic research cannot be understated.

Existing picture databases, however, have several limitations: usually, the number of items included in the data sets is relatively small; the most commonly used items are black and white line drawings (which when used in experiments can be informative only about a part of human visual processing); the response data are limited to response times; and most of the picture databases have norms in a single language. In order to go beyond the current limitations, we developed an entirely new database of colour photographs, with audio naming response data, and norms on four attributes.

We opted for colour photographs, as these have been shown to influence cognitive naming processes at the initial stage of visual identification. In this regard, both Rossion and Pourtois (2004) and Bonin et al. (2019), who compared line drawings, pictures with added grey-level texture, and colourised images, demonstrated that colour information significantly contributes to accuracy and naming speed by approximately 100 ms.

Moreover, besides commonly collected norming information on familiarity, visual complexity, or name agreement, we chose to collect ratings of valence and arousal. This was motivated by the fact that the existing affective picture databases, e.g. the International Affective Picture System (Lang et al., 1997) or the Geneva Affective Picture Database (Dan-Glauser & Scherer, 2011), include only limited norming information on neutral images, which have the power to induce non-negative emotions. By including valence and arousal ratings of common everyday objects in the current study, we aimed at establishing a useful baseline comparison for images preliminarily defined as positive or negative.

Furthermore, the importance of providing reliable norms on familiarity, visual complexity, valence, and arousal relates to the fact that the four variables have also been shown to influence image processing. That is, familiarity has been reported to correlate negatively with naming speed (e.g. Johnston et al. (2010) reported *r* = −.433). Regarding visual complexity, Snodgrass and Vanderwart (1980) and Rossion and Pourtois (2004) noted that a higher degree of image complexity might slow down image processing. However, this finding has not been confirmed by Perret and Bonin (2019) in their Bayesian meta-analysis. Finally, the impact of affective variables on image is well established. For instance, according to the Automatic Vigilance Hypothesis (Pratto & John, 1991), negative stimuli lead to delayed disengagement and thus, slower responses in recognition tasks (Estes & Adelman, 2008).

In what follows, we first review the existing picture data sets. Next, we move on to a discussion of the relevant megastudies which inspired the methodology used in this project. Finally, we present the experimental tasks that were administered as well as the initial findings established on the basis of the German data.

## Picture data sets

The rise in popularity of pictures as a research tool in psycholinguistics has not been matched by an increase in the quantity and quality of available stimuli. Many studies still rely on the black and white line drawings that were first developed by Snodgrass and Vanderwart (1980). This set of pictures with norms for naming agreement, image agreement, familiarity, and visual complexity consists of just 260 images. A salient characteristic of these pictures is that they are black and white drawings, which may be processed differently than images that are more realistic. The images from Snodgrass and Vanderwart (1980) were given a makeover by Rossion and Pourtois in 2004. They were coloured and a new archive, which includes 24-bit colour images of 209 objects, was created. In addition, normative data regarding the same four variables as in the original investigation were included. The comparison of the two data sets allowed Rossion and Pourtois (2004) to demonstrate that black and white line drawings attract lower recognition rates in comparison to colour images. Despite the quality of the pictures having been improved, the number of images in Rossion and Pourtois’ set is still relatively small.

An alternative set of pictures providing a more realistic and ecologically valid representation of real-life objects was created by Moreno-Martínez and Montoro (2012). It consists of 360 high quality colour images that belong to 23 semantic subcategories, e.g. fruit, animals, vehicles, clothes, etc. The normative data include information about age of acquisition, familiarity, manipulability, name agreement, typicality, and visual complexity. Nevertheless, the norms were only collected in Spanish, and overall, the number of images is still quite low.

To address some of the limitations of the smaller data sets, the open source Multilingual Picture (MultiPic) database (Duñabeitia et al., 2018) was recently released with 750 drawings that were normed across six languages, including British English, Spanish, French, Dutch (from Belgium and the Netherlands), Italian, and German. Over 600 native language speakers were requested to name the pictures (in typing) and rate their visual complexity on a Likert scale. The researchers established a high degree of convergence for naming in both within- and between-language conditions. Currently, however, MultiPic provides two norms and includes colour drawings of objects, which again restricts their usability in experimental settings.

In our view, the most comprehensive database of pictures that is currently available is the Bank of Standardised Stimuli (BOSS), with norms in American English (Brodeur et al., 2010; Brodeur et al., 2014) as well as a subset of items available in Canadian French (Brodeur et al., 2012). BOSS includes 1410 photo stimuli normed for name, semantic category, familiarity, visual complexity, object agreement, viewpoint agreement, and manipulability. Furthermore, the images are available in several versions, including greyscale, blurred, scrambled, and line drawings. This large set of images is an excellent source of experimental stimuli, but it is currently limited to two languages.

Finally, it is important to acknowledge the state-of-the-art platforms in object recognition, such as the Microsoft COCO: Common Objects in Context database (Lin et al., 2014) or the ImageNet database (Deng et al., 2009). They contain millions of annotated entries with images of varied quality embedded in the context of a visual scene. Certainly, in comparison to COCO or ImageNet, the current study and the LinguaPix database are small-scale. However, the fact that images in the two databases are embedded in a context and are of varying quality is very useful for artificial image recognition, although this makes them less appropriate for experimental research.

## Megastudy as a research tool

In the current study, 64,000 audio responses were recorded in German and the speech onset times (SOT) of these responses have been made available in the database. The quantity and scope of collected response data, in conjunction with its purpose of maximising utility and reusability, would qualify this as a megastudy (Keuleers & Balota, 2015; Keuleers & Marelli, 2020).

Seidenberg and Waters (1989) were the first to use the term *megastudy* to refer to the voice onset times that they collected based on 3000 monosyllabic English words. The studies that followed substantially increased the number of stimuli and the amount of data being collected. One of the first important examples of a megastudy was the English Lexicon project (Balota et al., 2007), which involved compiling lexical decision and naming data for over 40,000 words. This initial investigation gave rise to a number of variants: the French Lexicon project (Ferrand et al., 2010), the Malay Lexicon project (Yap et al., 2010), the Dutch Lexicon project (Keuleers et al., 2010), and the British Lexicon project (Keuleers et al., 2012), each providing data about several thousand words and pseudowords. The megastudy approach, however, has not just been limited to word recognition. In recent years, the approach has been applied to semantic priming (Hutchison et al., 2013), masked priming (Adelman et al., 2014), and even the processing of sentences by monolingual and bilingual speakers (GECO database by Cop et al., 2017). For the present study, the number of stimuli (1620) was comparatively small, but the responses elicited from 40 German-speaking participants resulted in a very large data set of audio files, and thus we have grounds to classify it as a megastudy. Before the data set is presented, the overall aims and the methodology used are described in the section below.

## Present study

The present study has the aim of addressing the limitations of the above-discussed picture data sets. Not only are many images, in the form of colour photographs, evaluated, but also—and importantly—the audio recordings of the naming data are used to establish SOT. The naming data are also used to derive the measures of naming agreement and accuracy. Finally, the rating data regarding familiarity, visual complexity, valence, and arousal are used to establish four linguistic norms. The resulting database of pictures, audio recordings, and linguistic norms will serve as a resource for the psycholinguistic research community.

## Method

### Participants

A group of 40 German native speakers took part in the study, all being university students between the ages of 18 and 26 (*M =* 22.2, *SD =* 2.8). The majority (29) were female. They were born in Germany and resided in this country at the time of the data collection. For all of them, German was their first and native language; however, they all spoke at least one foreign language. In addition, 15 of them reported speaking two foreign languages fluently, and four reported having knowledge of three.

### Stimuli

**Fig. 1:**
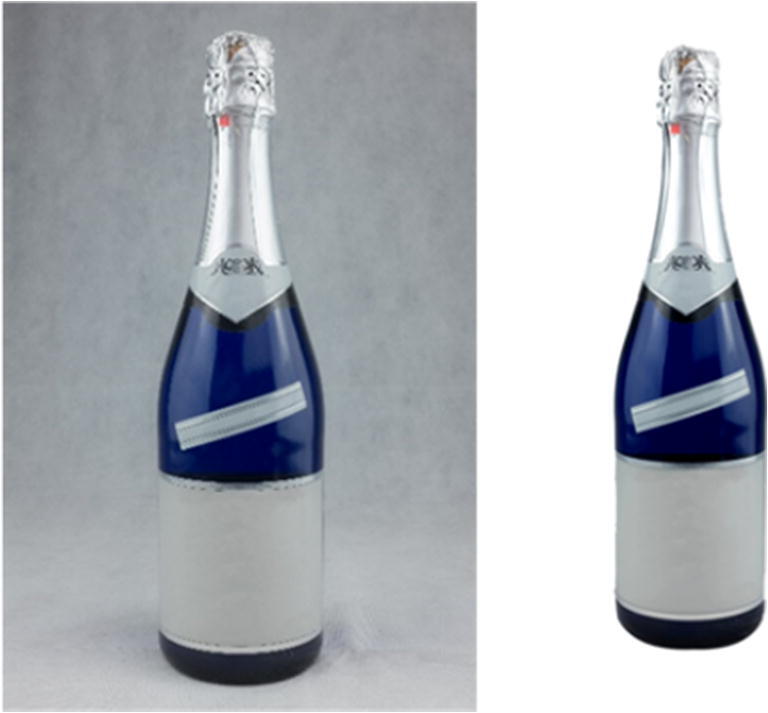
Illustration of the photo-editing process, with text having been already removed

**Fig. 2:**
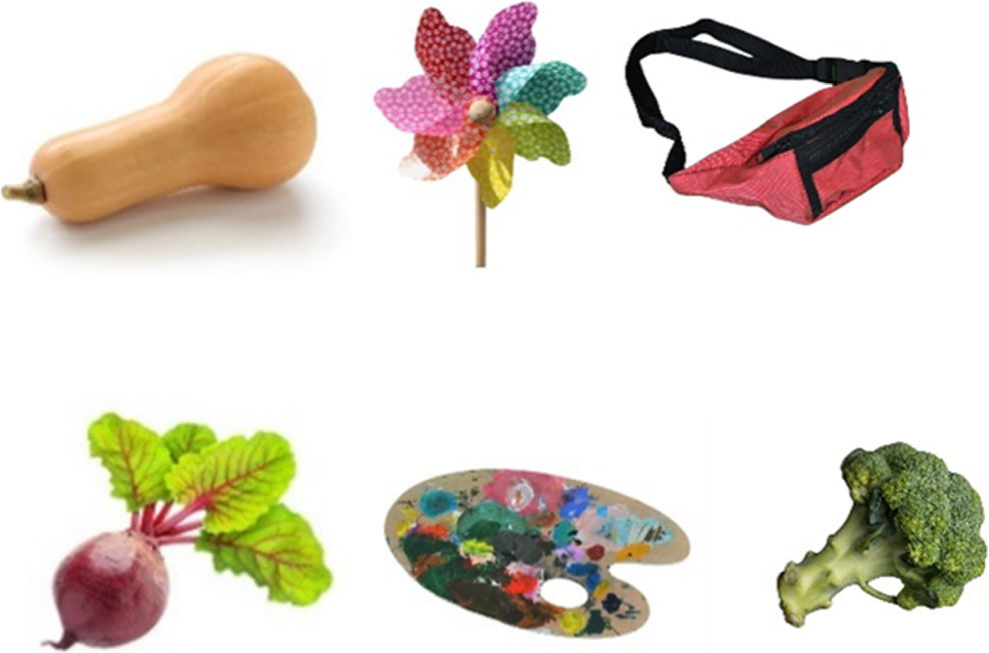
Examples of image stimuli

The initial stage of stimulus preparation involved creating lists of items from different semantic categories that could be photographed. We opted for stimuli that were concrete and imageable. Abstract notions, actions, and properties were initially considered, but were not included in the final list due to difficulties in capturing such items in a photograph. We arrived at over 1600 items spanning across 42 semantic categories including, inter alia, animals, plants, toys, professions, musical instruments, food, furniture, clothing and accessories, vehicles, buildings, stationery, and mythical creatures (Table 1). Next, over several months a student photographer took photos of the requested items. Each object was photographed on its own on a homogenous background, either green or white, at a resolution of 300 dpi. Subsequently, each photograph was edited. First, the ClippingMagic tool (https://clippingmagic.com) was used to remove the initial background and situate the object on a consistent white background. Then, the GIMP image editor (https://www.gimp.org/) was employed to remove any visible brand names, logos, or text, adjust the light, and resize the images. Part of the photo editing process is depicted in Fig. 1. The above-described procedure resulted in an initial set of 1220 photographs, examples of which are included in Fig. 2. It was not possible, however, to photograph several target items, e.g. different animals, sea creatures, or fairy tale characters. To address this issue, we purchased a set of 400 images from 123Rf (https://www.123rf.com/), which is a stock photo provider. To ensure the highest level of copyright protection, the legal department of the university, where the data were collected, drew up an individualised agreement with the image provider. The final list of items included 1620 photographs.

**Table 1:** List of the semantic categories and the numbers of items within each category that were photographed, including information about the main variables. Mean values and *SD*, in brackets, on a 6-point-Likert scale are given for familiarity, visual complexity, valence, and arousal. Accuracy and name agreement are presented in percentages

No.	Semantic category	No. of photos	Familiarity	Visual complexity	Valence	Arousal	Accuracy	Name agreement
1	Animals	46	4.88 (0.43)	3.52 (0.27)	4.27 (0.73)	3.43 (0.49)	93	89
2	Animal body parts	4	4.54 (0.48)	3.32 (0.18)	3.79 (1.07)	3.04 (0.67)	95	73
3	Bathroom appliances	23	5 (0.49)	2.74 (0.35)	3.64 (0.58)	2.36 (0.46)	81	90
4	Beauty products and tools	53	4.56 (0.68)	2.77 (0.44)	3.44 (0.45)	2.36 (0.32)	77	84
5	Beverages	19	5.08 (0.38)	2.54 (0.25)	4.22 (0.36)	2.98 (0.42)	81	56
6	Birds	19	4.84 (0.51)	3.61 (0.41)	4.17 (0.63)	3.18 (0.49)	88	83
7	Body parts	51	5.14 (0.28)	3.19 (0.33)	3.45 (0.46)	2.49 (0.44)	84	75
8	Buildings	17	4.13 (0.69)	3.18 (0.58)	3.85 (0.47)	2.58 (0.41)	78	81
9	Celebrations	18	4.84 (0.33)	3 (0.43)	4.42 (0.39)	3.2 (0.55)	86	86
10	Clothing and accessories	104	4.73 (0.57)	2.73 (0.41)	3.69 (0.45)	2.46 (0.46)	88	80
11	Colours	68	3.67 (0.61)	2.21 (0.32)	3.18 (0.49)	2.14 (0.44)	69	35
12	Electronic appliances	56	4.69 (0.72)	2.96 (0.54)	3.77 (0.51)	2.45 (0.5)	84	85
13	Flowers	26	4.68 (0.52)	3.02 (0.38)	4.28 (0.53)	3.08 (0.44)	77	53
14	Food	100	4.73 (0.72)	2.87 (0.43)	4.01 (0.56)	2.96 (0.59)	82	79
15	Fruit	49	4.79 (0.78)	2.79 (0.47)	4.3 (0.72)	3.19 (0.67)	78	80
16	Furniture	32	4.8 (0.5)	2.78 (0.35)	3.88 (0.5)	2.54 (0.61)	83	86
17	Games and toys	55	4.72 (0.51)	2.89 (0.58)	4.05 (0.51)	2.8 (0.55)	87	88
18	Garden tools	12	4.55 (0.51)	2.87 (0.34)	3.61 (0.53)	2.39 (0.53)	75	84
19	Home furnishings	41	4.49 (0.59)	3.14 (0.63)	3.79 (0.38)	2.47 (0.29)	82	78
20	Household chores	14	5.1 (0.49)	2.66 (0.33)	3.07 (0.32)	2.39 (0.31)	79	84
21	Household Items	63	4.56 (0.66)	2.79 (0.52)	3.49 (0.64)	2.47 (0.59)	81	83
22	Insects	15	4.71 (0.6)	3.22 (0.36)	3.04 (1.19)	3.54 (0.66)	91	87
23	Jewellery	12	3.98 (0.89)	3.3 (0.45)	3.6 (0.45)	2.45 (0.43)	74	83
24	Kitchen utensils	157	4.79 (0.59)	2.65 (0.47)	3.82 (0.44)	2.42 (0.5)	78	83
25	Marine life	10	4.47 (0.34)	3.36 (0.25)	3.72 (0.81)	3.29 (0.33)	94	91
26	Materials	11	3.83 (0.49)	3.06 (0.4)	3.55 (0.35)	2.14 (0.35)	76	75
27	Medical accessories	15	4.5 (0.63)	2.83 (0.42)	2.98 (0.71)	2.74 (0.52)	82	87
28	Musical instruments	29	4.32 (0.74)	3.38 (0.5)	4.12 (0.45)	2.86 (0.59)	76	80
29	Mystical creatures	4	4.04 (0.22)	3.85 (0.15)	3.34 (0.42)	2.86 (0.36)	90	95
30	Nature	14	4.76 (0.42)	3.24 (0.68)	4.24 (0.93)	3.23 (0.88)	87	86
31	Nuts	25	4.5 (0.77)	2.75 (0.39)	3.93 (0.56)	2.69 (0.5)	71	67
32	Parts of a house	20	4.64 (0.82)	2.81 (0.6)	3.69 (0.43)	2.27 (0.44)	83	86
33	Professions	28	4.6 (0.42)	3.56 (0.4)	3.71 (0.62)	2.78 (0.45)	93	89
34	Repositories	11	4.55 (0.63)	2.51 (0.43)	3.39 (0.62)	2.24 (0.6)	88	85
35	Shapes	28	3.99 (0.56)	2.02 (0.45)	3.37 (0.5)	1.93 (0.52)	64	67
36	Sports equipment	47	4.53 (0.61)	2.87 (0.42)	3.75 (0.48)	2.59 (0.43)	85	87
37	Stationery	61	4.81 (0.57)	2.61 (0.54)	3.64 (0.29)	2.13 (0.38)	79	84
38	Tools	61	4.33 (0.69)	2.7 (0.5)	3.36 (0.26)	2.12 (0.28)	73	85
39	Trees	6	4.65 (0.43)	2.87 (0.41)	4 (0.57)	3.06 (0.64)	87	53
40	Vegetables	69	4.65 (0.69)	2.8 (0.42)	3.88 (0.45)	2.68 (0.47)	72	79
41	Vehicles	41	4.61 (0.59)	3.41 (0.63)	3.87 (0.53)	2.72 (0.52)	87	91
42	Weapons	13	3.88 (0.37)	3.03 (0.48)	2.78 (0.64)	2.8 (0.56)	82	82

### Picture-naming experiment

Once the images had been prepared, we used them to design a picture-naming experiment. Stimulus display and recording of responses were performed using EPrime 2.0 software (Schneider et al., 2002). Given the large number of items that had to be named, the experiment was split into five smaller sub-experiments. Each experiment started with a short practice session, which included three items. Next, the experimental part began, whereby each image was presented individually on the screen in a randomised order for a duration of 3000 ms. The participants were instructed to provide a single word for each picture as soon as possible or to refrain from providing the name, if they could not recognise or were not familiar with the depicted object. They were also advised to avoid articles (e.g. *the apple*), adjectives (e.g. *green apple*), or full sentences (e.g. *It is an apple.).* Furthermore, since all responses were audio recorded, to extract the information about SOT, the participants were requested not to use hesitation devices (e.g. ‘hmmm’), cough, yawn, or sneeze. Detailed instructions that were given in the picture-naming experiments are included in Appendix 1.

### Online rating task

The rating task was designed using the online survey platform LimeSurvey (https://www.limesurvey.org/). Similar to the naming experiment, the questionnaire was split into five parts, with each individual item being presented with a set of four Likert scales ranging from 1 to 6 to evaluate familiarity, visual complexity, valence, and arousal. Familiarity was understood as the degree of how usual or unusual the presented item is in the realm of the participant’s experience (1 - *unfamiliar*, 6 - *familiar*). Visual complexity referred to the amount of detail or intricacy a given item depicts (1 - *very simple*, 6 - *very complex*). Valence pertained to the extent to which a given picture evokes positive or negative emotions in the participant (1 - *negative emotion*, 6 - *positive emotion*). Finally, arousal pointed to the intensity or strength of an emotion or an emotional state associated with a given picture (1 - *not intense*, 6 - *very intense*), which is similar to the definition of arousal by Warriner, Kuperman, and Brysbaert (2013, p. 1191). Whilst measures of arousal often use *calm* and *excited* as endpoints for the scale, we opted to use “keine intensive Emotion” (not intensive emotion), which captured calmness, and “sehr intensive Emotion” (very intensive emotion), capturing excitedness. Whilst researchers should be aware that the definition of arousal as degree of activation, from calm to exciting, is more commonly used (e.g. Bradley & Lang, 1999; Russell, 2003), our results also show the typical U-shaped relationship between valence and arousal (Fig. 5), which suggests that our operationalisation captures the same concept.

Our choice of a six-point rating scale diverges from the nine-point scale used by e.g. Lang et al. (1997), who have presented, arguably, the most influential collection of affective norms for pictures. At the same time, there is no uniformity in preceding research and there are no clear recommendations regarding the scales to use for collecting ratings for pictures and words. Next to the nine-point scale used for pictures and words (Bradley & Lang, 1999; Lang et al., 1997), the same authors used a 20-point scale for the computerised collection of affective ratings for words (Bradley & Lang, 1999). Five-point scales for familiarity, visual complexity, and image agreement are also common (Brodeur et al., 2012; Snodgrass & Vanderwart, 1980), and a 100-point scale has also been used for arousal, valence, and acceptability (Dan-Glauser & Scherer, 2011). It appears that most studies are guided by local research practices, rather than a clear convention. This lack of uniformity is not necessarily a drawback. In the end, ratings collected using different scales can always be compared by rescaling them or through standardisation. To increase the level of comparability of our data with other studies, we have also converted the six-point scales into five-point scales by following Cabitza (2015).

### Procedure

Prior to the data collection, each participant was presented with a consent form and detailed instructions about the study. The data collection for each participant took approximately six hours: two hours for the picture-naming experiments and four hours for the online rating tasks. The collection of the experimental data took place on the premises of the University of Mannheim, given the specialised software that had to be used as well as the need to audio record the responses. Each participant attended five naming sessions. In the online rating tasks, the items were presented sequentially, one at a time, with the four scales pertaining to the four variables. The participants were asked to rate the images rather than the concepts they portrayed. The original German instructions given in the rating task are included in Appendix 2. It was designed in such a way that the participants could navigate through the entire task by themselves. They could save parts of their responses and return to the task at a time or location convenient to them, as long as they had access to the Internet. For their effort and time, each participant was reimbursed €60 after completing all parts of the study.

## Results

From the 40 complete response sets, data from 38 participants were submitted for the final analysis. Data from two participants had to be removed as one of them had a very high percentage of incorrect responses or no responses given in the naming task. Data from the second participant contained lengthy and descriptive responses rather than actual naming of individual objects. Furthermore, three individual data sets, i.e. Ex 4 p. 118, Ex. 4 p. 134, and Ex. 3 p. 137, were not considered due to technical problems that occurred during the data collection process which prevented EPrime from saving the files correctly. Finally, the following items were removed, as a large proportion of participants found them especially difficult to name: *wine stopper* (20 speakers), *walking stick* (20), *tofu* (16), *soba noodles* (15), *seaweed* (16), *ring binding* (18), *razor* (17), *powder* (16), *pipe* (19), *pipe brush* (15), *pencil case* (17), *paper stand* (18), *paper clip* (19), *paper clip remover* (16), *milk frother* (17), *luggage scale* (15), *lemon peeler* (17), *inhalator* (15), *hinge* (18), *heater* (16), *fringe* (16), *fish and chips* (15), *durian* (10), *dragon fruit* (16), *diablo* (15), *couscous* (17), *cone* (18), *cocktail stirrer* (18), *clips* (18), *chisel* (15) and *camping gas* (15). The lack of responses in these cases might have been related to genuine unfamiliarity with the item or difficulties in recognising it due to problems with its depiction, e.g. an image of tofu. All further analyses were performed on the truncated data.

### Name agreement and accuracy

**Fig. 3:**
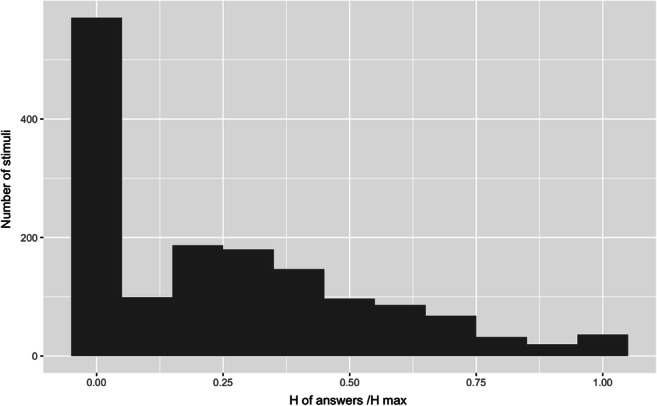
The distribution of H of answers divided by the H max

To establish the measure of name agreement and accuracy, we drew a random sample of the audio data from 10 participants from each experiment (16,000 .wav files), which were then manually transcribed and coded by two research assistants. The following two codes were used: 1 stood for a correct and complete word and 0 was entered for incorrect answers, incomplete ones, or no answer. Synonyms, near-synonyms (e.g. *Klebeband* or *Kreppband* for *adhesive tape*), and the superordinate of the category (e.g. *flower* instead of *rose*) were accepted as correct. This information allowed computing of the modal name for each image, which was the most frequently reported name for a particular image. That is, if the name agreement value was equal to 80%, eight participants out of ten (based on the amount of transcribed data) had provided the same word for the image. In many cases, however, two target names were most prominent and therefore, both were included in the database as a target name and an alternative one. The overall level of name agreement between the participants was relatively high; it was equal to 79% (± 23%). This level of name agreement is higher than that, for example, reported in the BOSS databases, standing at 64% for the first set and 59.5% for the second. The level we elicited resembles the information from normative data sets of line drawings that reported agreement between 72% and 85% (Bates et al., 2003). Next, entropy (*H*) was calculated on the probability distribution of alternative names. On average*,* normalised entropy was 0.69 (SD = 0.70), reflecting a relatively high level of naming agreement between the German participants. Reported levels mirror those reported, for example, by Snodgrass and Vanderwart (1980) 0.56 (±0.53) or Bates et al. (2003) from 0.67 (±0.61) to 1.16 (±0.79). Because *H* increases with the number of alternatives supplied, which crucially depends on the number of participants, we also included a normalised entropy measure, in which *H* is divided by the maximum entropy (*H*_*max*_) for a given number of alternatives, as shown in the equation below. A histogram capturing the distribution of normalised entropy is shown in Fig. 3.

$$ \frac{H}{H_{max}}=-\sum \limits_{i=1}^n\frac{p\left({x}_i\right){\log}_b\left(p\left({x}_i\right)\right)}{\log_b(n)} $$

The accuracy refers to the proportion of correct responses provided for each photograph. For example, an image of a hand mixer elicited the following labels: *Handmixer*, *Mixer*, *Handrührer*, or *Handrührgerät*; all of which were considered correct, but only the most frequently used ones were treated as the modal names, in this case the *Handmixer* and *Handrührer*. The accuracy rate across the final 1547 images was equal to 80% (± 22%). The semantic category of shape and colour returned the lowest accuracy rates (64% and 69%, respectively), despite the fact that in the colour category, we also treated focal colour terms as correct. That is, the semantic category of colour comprised 70 unique hues presented as coloured stains. Hardly any participant made a distinction between peripheral terms, such as *crimson*, *ruby*, or *red*, but rather referred to all these shades as *red*, which was scored as a correct answer. The categories of nuts (71%), vegetables (72%), and tools (73%) also had relatively lower accuracy rates. On the other hand, the categories of insects, professions, animals, marine creatures, and vehicles returned above 90% accuracy.

### Familiarity, visual complexity, valence, and arousal ratings

**Fig. 4:**
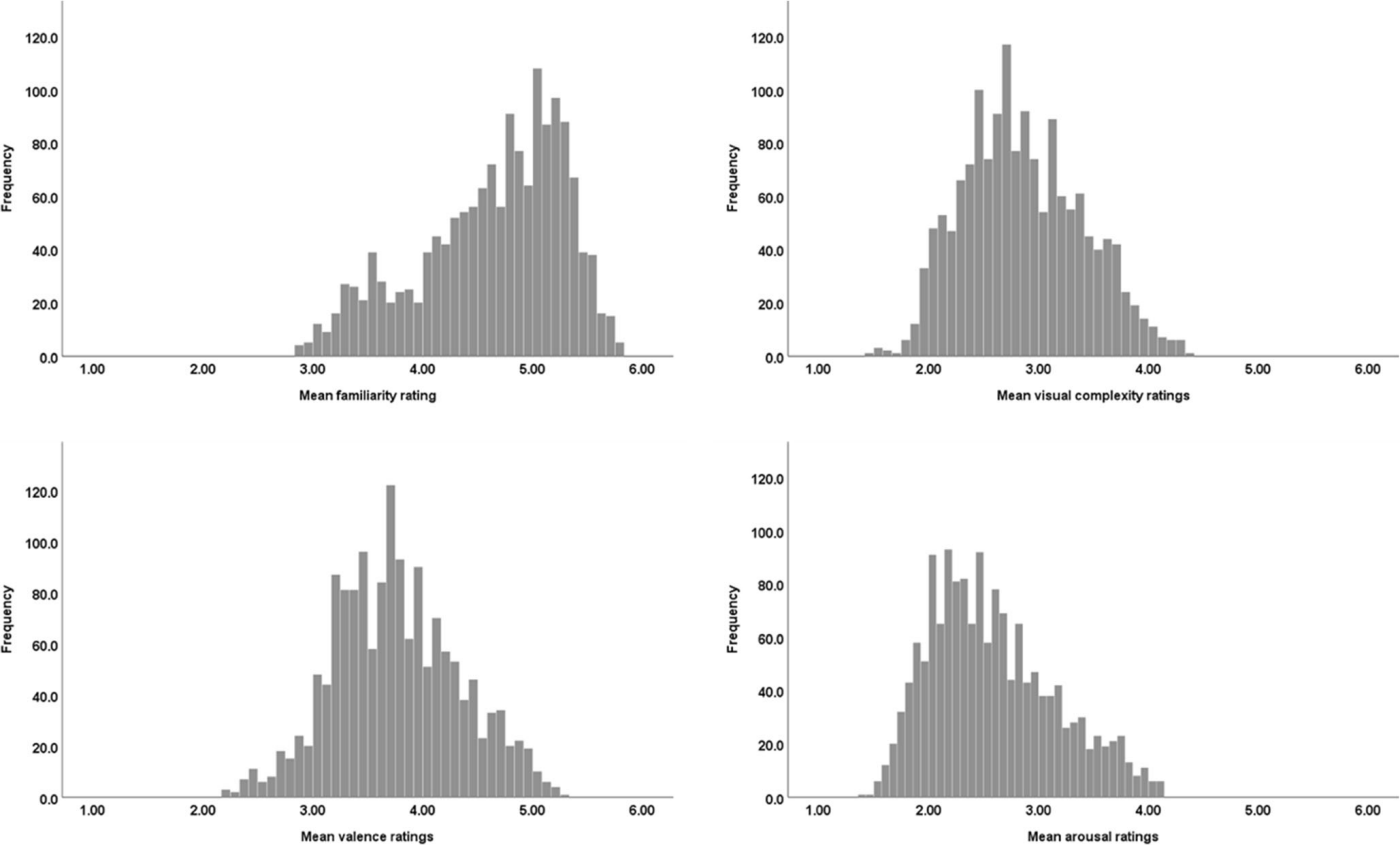
Distribution of mean familiarity, visual complexity, valence, and arousal ratings. The 1 to 6 scales correspond to: 1 - *unfamiliar*, 6 - *familiar*; 1 - *very simple*, 6 - *very complex*; 1 - *negative emotion*, 6 - *positive emotion*; 1 - *not intense*, 6 - *very intense*

The rating data on familiarity, visual complexity, valence, and arousal were aggregated across the participants and items. The overall distribution of each variable, excluding outliers comprising 0.5%, which were replaced with mean values, is presented in Fig. 4. The mean familiarity ratings were equal to 4.63 (*SD* = .02). Converted to a five-point scale, this becomes 3.9, which is higher than the score reported by Snodgrass and Vanderwart (1980), i.e. 3.3 (*SD* = 1.0), but closer to the average scores from the first BOSS database, i.e. 4.0 (*SD* = .4) (Brodeur et al., 2010) as well as the second BOSS, 4.16 (*SD* = 0.55) (Brodeur et al., 2014). A Kolmogorov–Smirnov test for normality returned a statistically significant result, *D* = .09, *p* = .000, which does not confirm the normality of the data. The distribution was negatively skewed. The average visual complexity rating in our study was 2.86 (*SD* = .01). Converted to a five-point scale, this becomes 2.48, which is lower than that what was reported by Snodgrass and Vanderwart (1980), 3.0 (*SD* = .9) and similar to the mean ratings from BOSS parts one and two, i.e. 2.4 (*SD* = .4). A Kolmogorov–Smirnov test showed that the visual complexity variable was not normally distributed, *D* = .05, *p* = .000; it was positively skewed. The mean valence ratings were equal to 3.76 (*SD* = .01) and arousal ratings to 2.58 (*SD* = .01). Converted to a seven-point scale, the mean valence rating was 4.31 and the mean rating for arousal was 2.89, which allows for comparison to the Open Affective Standardised Image Set (OASIS) (Kurdi et al., 2017). They reported a similar mean value of 4.33 (*SD* = 1.10) for valence, but a higher mean value of 3.66 (*SD* = 1.68) for arousal. Two Kolmogorov–Smirnov tests performed on the variable of valence and arousal revealed that both factors are not normally distributed, *D*_*valence*_ = .04, *p* = .000 and *D*_*arousal*_ = .07, *p* = .000.

**Fig. 5:**
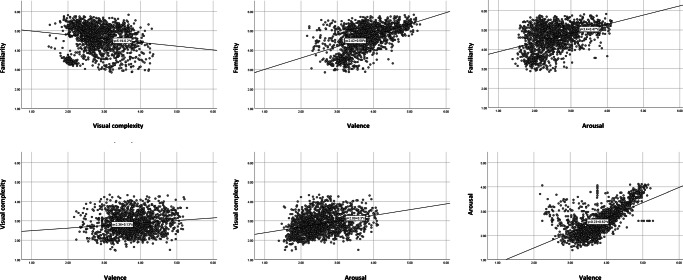
Relationships between familiarity, visual complexity, valence, and arousal ratings

As an estimate of the reliability of the average ratings for items, we computed the intraclass correlation coefficient ICC (*C, k*) for each of the variables (McGraw & Wong, 1996; Shrout & Fleiss, 1979). For all of the rated variables, reliability was high: 0.94 for familiarity, 0.89 for visual complexity, 0.92 for valence, and 0.90 for arousal. The relationships between all four variables are shown in Fig. 5. Furthermore, significant linear relationships were found between all the variables investigated. A weak but significant negative correlation was shown between familiarity ratings and visual complexity ratings, *r* = −.170, *p* = .000, implying that more familiar images are also less visually complex ones. This finding is confirmatory of what Snodgrass and Vanderwart (1980) demonstrated. Their analysis based on 260 line drawings returned a significant negative correlation of *r =* −.466. Furthermore, Pearson’s correlation between familiarity and valence as well as familiarity and arousal returned statistically significant positive correlations, respectively *r* = .508, *p* = .000 and *r* = .430, *p* = .000. This implies that photos that were judged as being more familiar were also seen as being more positive and more arousing. Next, the comparison of the visual complexity rating with valence and arousal proved to be statistically significant, with weak positive correlations reported in both cases, *r* = .134, *p* = .000 and *r* = .327, *p* = .000. More visually complex images were judged as being slightly more positive on the valence variable and more arousing. Finally, a moderate positive correlation can be seen between valence and arousal, *r* = .569, *p* = .000. Rather counterintuitively, images that are more positive were rated to be more arousing. This finding is in conflict with that reported by, for example, Kurdi et al. (2017), who showed the lack of a statistical relationship between valence and arousal, *r* = .06, *p* = .081. However, Warriner et al. (2013) found a positive correlation between arousal and valence for positive words and a negative correlation for negative ones. Since the proportion of negatively valenced photographs in the present data set is relatively small, the present finding could be attributed to undersampling of low-arousal positive and negative images.

### Rating scales, name agreement, and accuracy

An analysis of the four rating scales (familiarity, visual complexity, valence, arousal) and the name agreement and accuracy values returned statistically significant positive correlations at the 0.01 level (two-tailed) between all but one pair of factors, that of visual complexity and accuracy (*r* =−.013, *p* = .616). The correlation coefficients of the pairwise relations are given in Table 2 below. The results demonstrate that name agreement and accuracy were higher for those images that participants were familiar with, those that were visually more complex, as well as those that had evoked positive emotions of higher intensity.

**Table 2:** Correlation coefficients of the pairwise relations between all rating scales, name agreement and accuracy

	Accuracy	Name agreement
Familiarity	.412**	.264**
Visual complexity	−0.013	.077**
Valence	.194**	.113**
Arousal	.244**	.139**
Accuracy	1	.332**
Name agreement	.332**	1

**Correlation is significant at the 0.01 level (two-tailed)

### Speech onset times

**Fig. 6:**
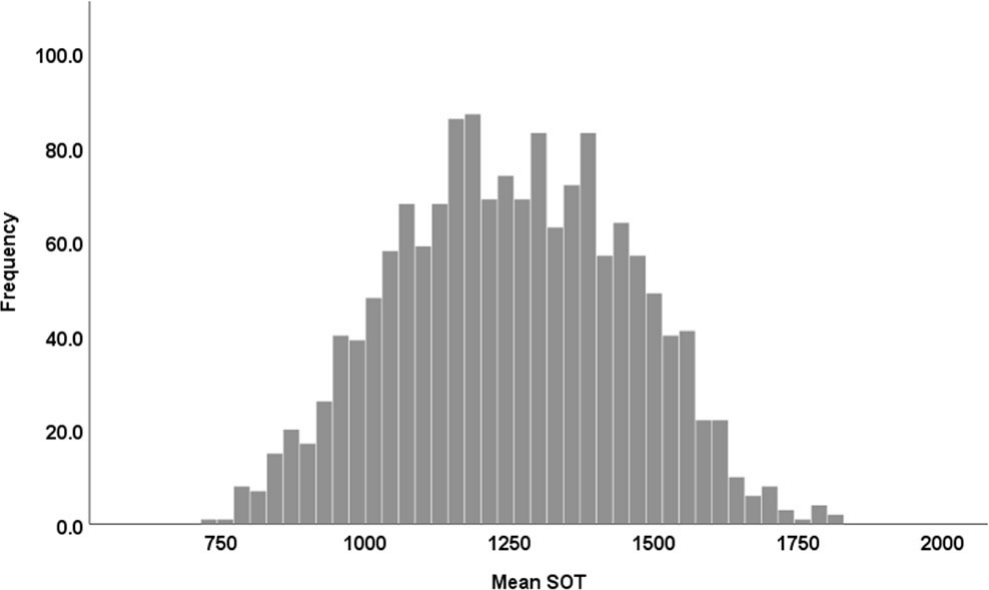
Distribution of mean SOT

The detection of SOT was performed with the automated Chronset tool (Roux et al., 2017). Before the SOT were analysed, the data were prepared in the following way. Responses outside of two standard deviations from the participant’s mean across all five naming experiments were treated as outliers and were removed from further analysis (5.6%). In addition, items that were not named (11%) and hence, produced no SOT, were not considered. This procedure allowed for establishing a mean naming speed per participant across the final 1547 images. The descriptive information regarding SOT is presented in Fig. 6. The average SOT across all participants and items was equal to 1252 ms (*SD* = 5.1) and the range varied from 718 ms to 1817 ms. The average response latencies are relatively slow, which might be reflective of the overall difficulty of the task. Participants named a large number of heterogeneous items, which for the most part are of medium to low frequency. Similar to the reliability of the average affective ratings for the photographs, the reliability of the average SOT for photographs was high: ICC (C, k) = 0.91.

**Fig. 7:**
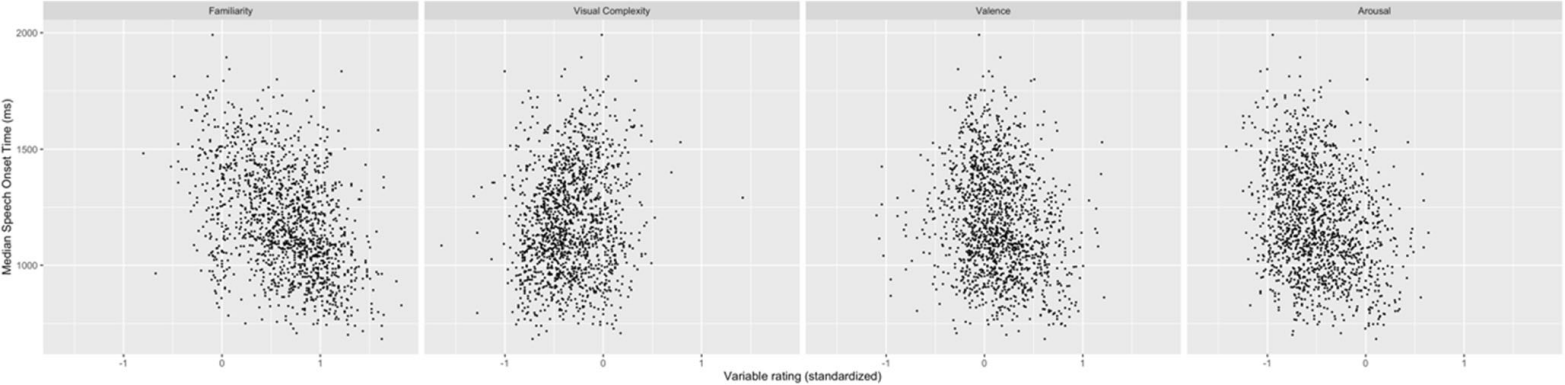
Relationships between familiarity, visual complexity, valence, arousal ratings, and SOT

The linear relationships between the standardised variable rating and the median SOT are given in Fig. 7. Negative correlations between SOT and familiarity, *r* = −.409, *p* = .000, valence, *r* = −.187, *p* = .000, and arousal, *r* = −.252, *p* = .000, illustrate that the participants took longer to respond to images that were less familiar, more negative, and less arousing. Furthermore, a weak positive linear relationship was present between SOT and visual complexity, *r* = .132, *p* = .000. This finding is to be expected as more visually complex images need slightly more time for processing. A review of previous studies on predictors of picture-naming speed, for example, by Alario et al. (2004, p. 146), demonstrates that the effect of concept familiarity and visual complexity on naming latencies is not consistently found across studies investigating different languages. However, when the effects are present, they follow the same patterns as those presented in the current investigation.

Next, an analysis of variance revealed a statistically significant effect of familiarity, visual complexity, valence, and arousal ratings as well as a two-way interaction between familiarity and visual complexity on SOT. However, the only notable effect size (*η*^2^) was that of familiarity, accounting for almost 10% of the variance (see Table 3).

**Table 3:** Analysis of variance showing the effects of predictors on SOT

Variable	Sum of squares	*df*	*F*	*p*	*η*^*2*^
Familiarity	6,770,723	1	170.72	0.00	0.0967
Visual complexity	408,220	1	10.29	0.00	0.0058
Valence	504,980	1	12.73	0.00	0.0072
Arousal	675,977	1	17.04	0.00	0.0097
Familiarity × Arousal	44,016	1	1.11	0.29	0.0006
Familiarity × Visual complexity	419,025	1	10.57	0.00	0.0060
Familiarity × Valence	126,815	1	3.20	0.07	0.0018
Visual complexity × Arousal	45,485	1	1.15	0.28	0.0007
Valence × Arousal	49,599	1	1.25	0.26	0.0007
Residuals	60,956,970	1537			

*Note:* The final column indicates the effect size (*η*^2^) for each term

## Discussion

**Fig. 8:**
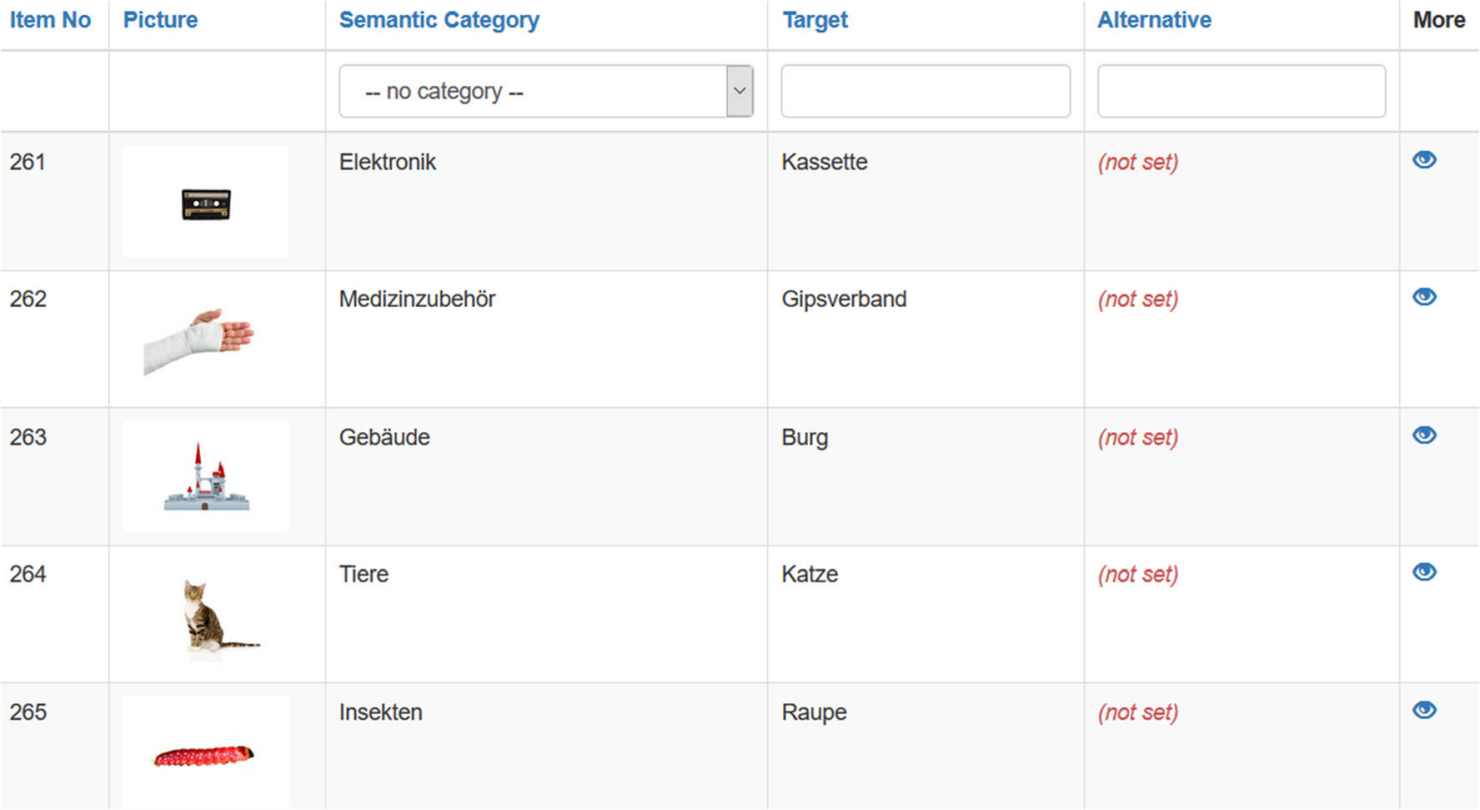
A screenshot of the main page of the LinguaPix database interface

**Fig. 9:**
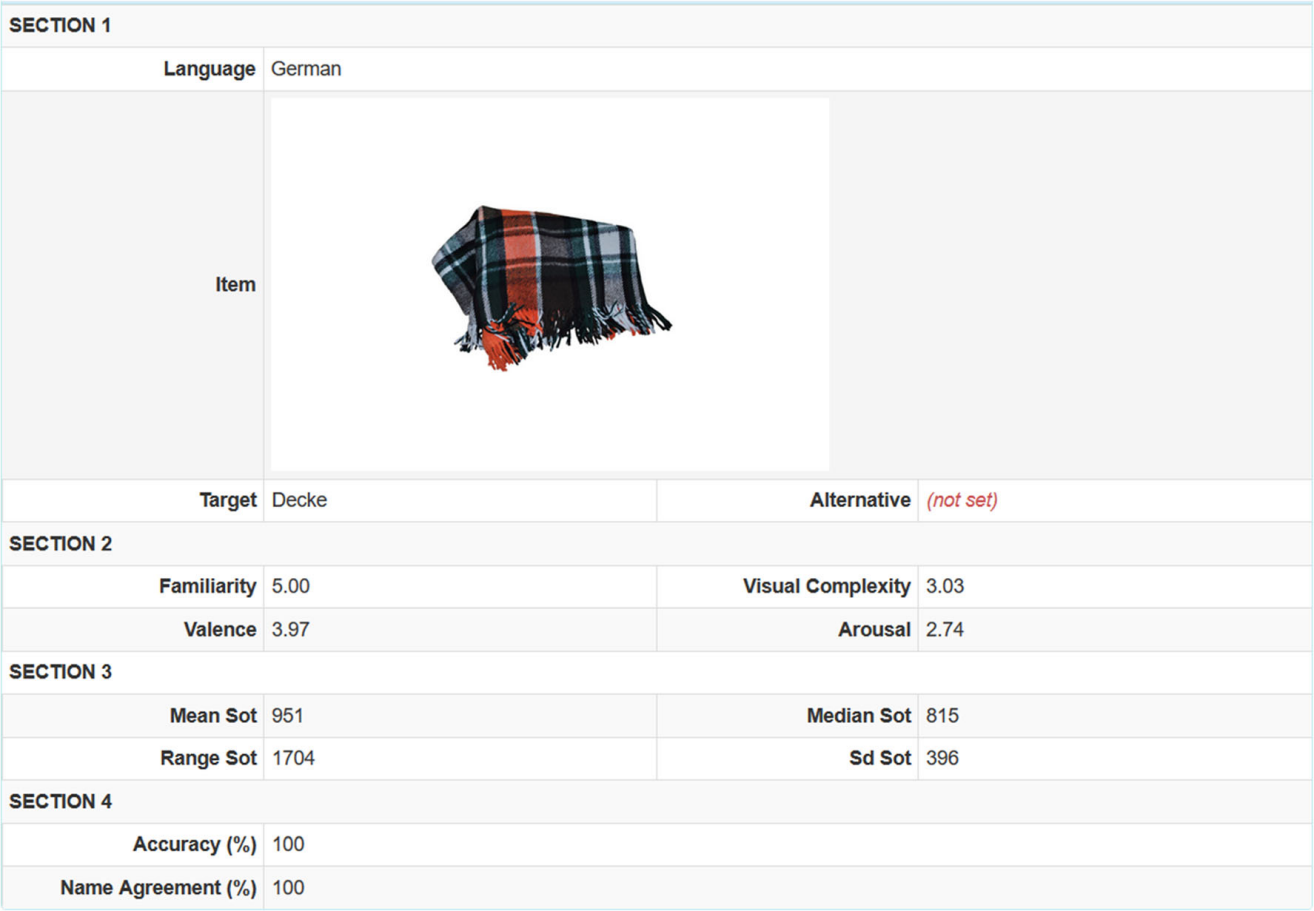
A screenshot of an image page of the LinguaPix database interface

To create the LinguaPix database (http://linguapix.uni-mannheim.de), we have taken and normed over 1600 colour images across the following variables: SOT, name agreement, accuracy, familiarity, visual complexity, valence, and arousal. In contrast to many previous studies, we did not request the participants to type the names of the images, but rather, to name them orally, which allowed for recording of SOT. The current version of the database, created on the basis of German data, comprises 1547 photographs from 42 semantic categories. The items along with the respective categories and target names are arranged alphabetically (Fig. 8). Each photograph, together with detailed information about it, including three examples of audio recordings, can be viewed and downloaded in a larger format (3540 × 2369 pixels) (Fig. 9). The remaining audio material for each photograph is available upon request. The photographs are searchable by item name, semantic category, or pre-specified criteria, e.g. familiar items or those that evoke negative emotions, by applying the advanced filter. Also, all data are accessible in CSV format on signing up to the database.

The uniqueness of the LinguaPix database lies in the fact that it contains a large number of colour images, extensive information about SOT, linguistic norms, and valid participant characteristics that can be used in future research. The planned extension of LinguaPix will create substantial economies of scale, as the photographs are already taken and edited. The scope of the database can be enlarged by adding naming norms from additional languages, including Dutch, English, Polish, and Cantonese.

The rating data and the SOT collected online and in the picture-naming experiment have revealed several interesting patterns. For instance, linear relationships were observed between all rating variables. Familiarity correlated in a negative way with visual complexity, but had a positive relationship with valence and arousal. The correlations between visual complexity and both valence and arousal turned out to be positive. Finally, when valence and arousal were compared, a positive relationship between the two variables was observed. In addition, the data from the analysis of variance revealed that all rating variables contributed to explaining SOT variance, albeit to varying degrees. Familiarity with the image was most discriminant of the SOT, followed by arousal and visual complexity, and valence to a lesser extent.

Our results show faster picture naming with increasing valence and arousal. In the case of valence, this pattern is consistent with findings presented by e.g. White et al. (2016), who reported slower naming for negative pictures. On the other hand, De Houwer and Hermans (1994) found no difference between positive and negative words in picture naming. In the few studies that have looked at the effect of valence and arousal on picture naming, Blackett et al. (2017) reported that both positive and negative pictures with high arousal were named slower than neutral stimuli with lower arousal.

To the extent that word naming and picture naming can be considered similar, our results for valence are compatible with the analysis of Kuperman et al. (2014), who re-analysed a series of influential studies (Estes & Adelman, 2008; Kousta et al., 2009; Larsen et al., 2008) and showed that, for words within the same frequency range, negative ones are recognised more slowly than positive ones. On the other hand, Kuperman et al. also found that less arousing words are recognised faster than more arousing ones, which is the opposite of the pattern we have demonstrated. These similarities and discrepancies invite more thorough analyses of our results.

The analysis reported in this manuscript is certainly not exhaustive. We focused mainly on presentation of the major relationships between the variables. Further analysis is planned that will (1) incorporate the demographic variables, (2) compare the cross-linguistic data from the additional four languages, and (3) contrast the available data sets from recognition of photographs with recognition of black and white line drawings, coloured drawings, and the recognition of words. Since line drawings often resemble prototypical representation and photographs are individualised depictions of items, a processing difference is to be expected. Finally, a comparison of the processing times of photographs and words can further aid the discussion regarding the visual and lexico-semantic stages of recognition.

We recognise several limitations that the current study faced. One of the issues relates to the experimental design and the fact that the images were presented on the computer screen for a duration of 3000 ms. In the case of infrequent or unusual items, participants did not manage to retrieve the name in the allowed time, which resulted in 11% of the SOT not being available. In addition, since the images were presented in a random order and the participants were not familiar with the range of items being depicted, this might have influenced the precision of their answers. That is, if, for example, an image of a *hazelnut* appeared first, it would often attract the name *nut*. Only when the participants came across *peanuts*, *Brazil nuts*, etc., did they start to differentiate between the names, despite the fact that they were instructed to be specific in naming. Finally, items such as *mustard*, *toothpaste*, *liquid soap*, *hair spray*, and *shaving foam* proved rather challenging to be named without any additional clue regarding the name of the product or the brand. Often *shaving foam* was referred to as *hair foam*, *hair spray* ended up being a *spray paint*, and *mustard* was simply named a *tube*.

Despite several caveats, we anticipate a variety of use cases for the data collected in this study, adding methodological variety and richness and thus, offering new avenues for research. A first area is replication: existing experiments for which picture-naming times were the dependent variable can be reanalysed using the SOTs to photographs from the current study. In a similar way, studies that have used ad hoc ratings for familiarity, visual complexity, valence, and arousal can be re-evaluated using the rating data collected here. A second area is the investigation of new research questions: instead of setting up an experiment to collect new data, researchers can check whether the data they would want to collect are already available. This applies to both the SOTs and the rating scales. A related application lies in stimulus selection for other fields, such as memory research. In the field of psycholinguistics, the data can also offer insights into the differences in processing photographic and pictorial representations of the same concepts. Finally, researchers in artificial intelligence may be interested in using the data to train picture-to-word recognition models or to train speaker identification ones.

## Conclusion

To address the shortcomings of the extant picture-naming databases, we have conducted a megastudy of picture-naming norms. A group of German native speakers named and evaluated over 1600 colour images on measures of familiarity, visual complexity, valence, and arousal. This allowed for establishing the norms of name agreement, accuracy, and gathering information about SOT. The resulting LinguaPix database is the largest available tool of its kind and it is currently being extended to four more languages: Dutch, English, Polish, and Cantonese. Since databases act primarily as resources, we see potential in applying information from LinguaPix in psycholinguistic research, cognitive psychology research, computational linguistics, i.e. training image recognition algorithms, or language learning and language impairment research, i.e. adapting the photographs into a digital diagnostic tool for receptive vocabulary comprehension with children or aphasic patients. Finally, we would welcome extending the database to other languages which are currently not under investigation.

## Appendix 1 - Instructions – picture-naming task

In this experiment, you will be requested to name pictures presented on the screen. One picture will be shown on the screen at a time. The items will change automatically once 5 seconds have elapsed. There is no need for you to press any buttons between the individual trials.

Please speak clearly. The microphone will automatically record all your answers in order to measure speech onset times and name agreement.

Important:

- don’t use articles (e.g. the apple) but name the item itself (e.g. apple)
- don’t use adjectives to describe the items (e.g. green apple) but (e.g. apple)
- don’t use hesitation devices (e.g. hmmm)
- don’t use full sentences (e.g. It is an apple) but singe words/nouns (e.g. apple)
- try to avoid coughing, yawning, sneezing, if possible
- the photographs of coloured ‘powder’ require you to produce the name of the colour
- be specific but use the first word that comes to your mind
- name the items as fast as possible
- if you don’t know or don’t remember the name of the item don’t say anything

If you have made a mistake, e.g. named an item incorrectly, do not worry, simply proceed with the task.

## Appendix 2 – Instructions – German online rating task

In der folgenden Umfrage bitten wir dich darum, jedes Bild anhand von vier Maßstäben zu bewerten: (1) Vertrautheit (wie üblich oder unüblich das Objekt, das im Bild präsentiert wird, für dich ist), (2) visuelle Komplexität (die Detailliertheit oder Kompliziertheit, die ein gegebenenes Bild darstellt), (3) emotionale Wertigkeit (in welchem Maße ein gegebenes Bild eine positive oder negative Emotion auslöst), (4) Erregung (die Intensität oder Stärke eines emotionalen Zustandes, der mit einem gegebenen Bild verbunden wird).
